# Correlation between flexural and indirect tensile strength of resin composite cements

**DOI:** 10.1186/s13005-016-0128-7

**Published:** 2016-11-04

**Authors:** Gianluca Cassina, Jens Fischer, Nadja Rohr

**Affiliations:** Division of Dental Materials and Engineering, Clinic for Reconstructive Dentistry and Temporomandibular Disorders, University Hospital of Dental Medicine, University of Basel, Hebelstrasse 3, 4056 Basel, Switzerland

**Keywords:** Resin composite cement, Curing mode, Auto-polymerizing cement, Dual-curing cement, Flexural strength, Indirect tensile strength, Aging

## Abstract

**Background:**

To evaluate a potential correlation between flexural strength and indirect tensile strength in assessing the mechanical strength of resin composite cements.

**Methods:**

Flexural strength (*n* = 5) and indirect tensile strength (*n* = 5) of 7 resin composite cements (RelyX Unicem 2 Automix [RXU], Panavia SA [PSA], Clearfil SA [CSA], Panavia F2.0 [PF2], Multilink Implant [MLI], DuoCem [DCM], Panavia 21 [P21]) were determined. Specimens were either auto-polymerized or dual-cured (except P21) and stored in water at 37 °C for 1 day prior to measurement. Flexural and indirect tensile strength of 4 cements (RXU, PSA, PF2, MLI) was additionally measured directly after curing and after 96 h water storage at 37 °C.

**Results:**

Except for PF2, dual-cured specimens achieved higher flexural strength than auto-polymerized specimens. In the indirect tensile strength test differences in auto-polymerized and dual-cured specimens were only detected for RXU and DCM. A general non-linear correlation was found between flexural and indirect tensile strength values. However, strength values of auto-polymerized and dual-cured specimens did not generally correlate.

**Conclusions:**

Flexural strength and indirect tensile strength of resin composite cements are correlated. At high strength values the indirect tensile test is less sensitive than the flexural test. The results suggest that the indirect tensile test may only be recommended as a screening test especially for low or medium strength resin composite cements.

## Background

The restoration of teeth with ceramic crowns is an important aspect in oral rehabilitation. In order to provide an aesthetic outcome, silicate ceramics are the material of first choice [1]. However, their mechanical strength is low [2]. The mechanical strength of silicate ceramic restorations is strongly influenced by the mechanical properties of the cement [3]. Therefore, assessing the mechanical strength of resin composite cements is important to classify these materials.

In the last decades there was a strong improvement of dental luting agents. Consequently a wide range of possibilities for the cementation of fixed restorations is offered to the clinician nowadays. Resin composite cements may be polymerized by auto-polymerization or by light-activation [4]. Light-curing starts with the exposure to a light within a defined wave-length, activating the initiator, thus starting the polymerization. Auto-polymerization requires two components, one containing the activator, the second one the initiator. Polymerization starts immediately after mixing. Campherquinone is generally used as an initiator for light activation, benzoyl peroxide for auto-polymerization [5]. Inhibitors are added to delay the polymerization process. Thus sufficient time is available to place the restoration. Today, most resin composite cements provide both activation systems allowing the placement of a restoration within sufficient time while guaranteeing complete polymerization. To assess the clinical performance of these cement materials, in vitro tests prior to clinical application are required.

Resin composite cements are widely tested for their bonding capacities, but mechanical strength tests are rare [6, 7]. Mechanical strength is assessed by flexural strength. However, the preparation of the test specimens is time consuming and demanding. The preparation of test specimens for indirect tensile or compressive strength test is easier. Further, the indirect tensile strength proved to be more sensitive than a compressive strength test [8]. In that study, the indirect tensile test revealed to be an appropriate test to assess the influence of artificial aging on a resin composite cement: A decrease in indirect tensile strength was observed for water storage at 37 °C as well as thermocycling over an aging period of 64 days for a conventional resin composite cement.

The purpose of this study was to investigate if indirect tensile strength test and flexural strength test of resin composite cements provide similar results.

## Methods

Seven resin composite cements were used in this investigation, 3 self-adhesive and 4 conventional resin cements (Table 1). Flexural and indirect tensile strength tests were performed on dual-cured and auto-polymerized cement specimens after 24 h water storage. Further, with 2 self-adhesive and 2 conventional resin composite cements (RXU, PSA, PF2, MLI) measurements were performed immediately after curing as well as after 96 h water storage at 37 °C.

**Table 1:** List of resin composite cements used

Material	Code	Type	Manufacturer	Lot No.
RelyX Unicem 2 Automix	RXU	self-adhesive, dual-curing	3 M ESPE Seefeld, Germany	585065
Panavia SA	PSA	self-adhesive, dual-curing	Kuraray Noritake Dental Kurashiki, Japan	069ABA
Clearfil SA	CSA	self-adhesive, dual-curing		0057 AA
Panavia F2.0	PF2	conventional dual-curing		A:0575A/B:0289B
Multilink Implant	MLI	conventional dual-curing	Ivoclar VivadentSchaan, Liechtenstein	RS 6681
DuoCem	DCM	conventional dual-curing	Coltène Whaledent Altstätten, Switzerland	679895
Panavia 21	P21	conventional auto-curing	Kuraray Noritake Dental Kurashiki, Japan	41407

### Specimen preparation for flexural strength tests

For flexural strength measurements specimens with dimensions of 25.0 x 2.0 x 2.0 mm were produced according to ISO 4049:2009 in sets of 10. Cements were mixed according to the manufacturer’s instructions and filled into cavities of a customized Teflon mold. Glass slides of 1 mm in thickness covered with a transparent Mylar foil (Hawe Transparent Strips, KerrHawe, Bioggio, Switzerland) were placed on both sides of the mold to keep the cement in place. Five specimens of each set were light cured, 5 specimens were auto-polymerized.

For auto-polymerization 5 specimens were left in the mold for 60 min in a dark box. The remaining 5 specimens were light-cured with a polymerization lamp (Elipar, 3 M ESPE, Seefeld, Germany) with an intensity of 1200 MW/cm^2^. The exit window of the LED lamp had a diameter of 8 mm. It was positioned directly on the glass slide, thus maintaining a well-defined distance of 1 mm to the specimen’s surface. Light curing started in the center of the specimen. After light exposure the exit window was moved to the section next to the center overlapping the previous section by half the diameter of the exit window (i. e. 4 mm). The procedure was repeated until the specimen on the one side of the center had been completely irradiated. Thereafter the section on the other side of the center was light-cured the same way. The procedure described was repeated from the rear side of the specimen. Duration of each light exposure was 20 s. After light-curing was completed, the cements were left in the mold for 15 min. Specimens were carefully removed from the mold and deflashed with 320 grit sandpaper (3 M, St. Paul, MN, USA). All specimens were stored in water at 37 °C for 24 h.

### Specimen preparation for indirect tensile strength tests

To measure indirect tensile strength, cylindrical test specimens (3 mm in height and 3 mm in diameter) were produced in sets of 10 using a customized Teflon mold. The procedure was similar to that applied for flexural strength specimens. Light-curing was done by irradiating both sides for 20 s each.

### Measurement of flexural strength

Flexural strength measurements were performed according to ISO 4049. Height and width of each bar was measured in the middle of the specimen with a digital caliper (Mitutoyo 500-181-30, Mitutoyo Europe, Neuss, Germany). To measure the flexural strength the specimens were positioned on two cylindrical supports with diameters of 2 mm. The distance between the supports was 20 mm. Loading was performed with a cylindrical loading piston with a diameter of 2 mm in the middle of the bar, using a universal testing machine (Z020, Zwick/Roell, Ulm, Germany). The crosshead speed was set to 1 mm/min. Load at fracture was registered and the flexural strength (σ_FS_) was calculated according to the following equation:

$$ {\upsigma}_{\mathrm{FS}} = 3\mathrm{F}\mathrm{l}/2{\mathrm{bh}}^2 $$

F: load at fracture

l: distance between the supports (20 mm)

b: width of the specimen

h: height of the specimen

### Measurement of indirect tensile strength

Height and diameter of the specimens were measured and the specimens were loaded perpendicular to the cylinder axis until fracture (Z020, Zwick/Roell). Load at fracture was registered and the indirect tensile strength (σ_ITS_) was calculated according to the following equation:

$$ {\upsigma}_{\mathrm{ITS}} = 2\mathrm{F}/\left(\uppi \mathrm{d}\mathrm{h}\right) $$

F: load at fracture

d: diameter of the specimen

h: height of the specimen

### Effect of water storage

Additional light-cured and auto-polymerized specimens of RXU, PSA, PF2 and MLI (*n* = 5) were manufactured to assess the effect of water storage. Flexural strength and indirect tensile strength were measured immediately after curing and after 96 h water storage at 37 °C.

### Statistics

All data was analyzed with Shapiro-Wilk normality test. Statistical analysis was performed with one-way ANOVA for normal distributed data and with Kruskal-Wallis test for not normal distributed data (*p* < 0.05). Statistic software StatPlus:mac Pro Version v6 (2016), AnalystSoft Inc. was used.

## Results

### Flexural strength tests

For all materials except PF2 a significant higher flexural strength was recorded for dual cured specimens compared to the auto-polymerized specimens after 24 h water storage (Table 2). Shapiro-Wilk test revealed a normal distribution for flexural strength data, hence one-way ANOVA was applied to test for differences between the cements. For auto-polymerization MLI achieved highest values followed by DCM, PF2, P21, PSA, CSA, and RXU. In the dual-cured state specimens of MLI revealed highest flexural strength values, followed by DCM, RXU, PF2, CSA, and PSA.

**Table 2:**
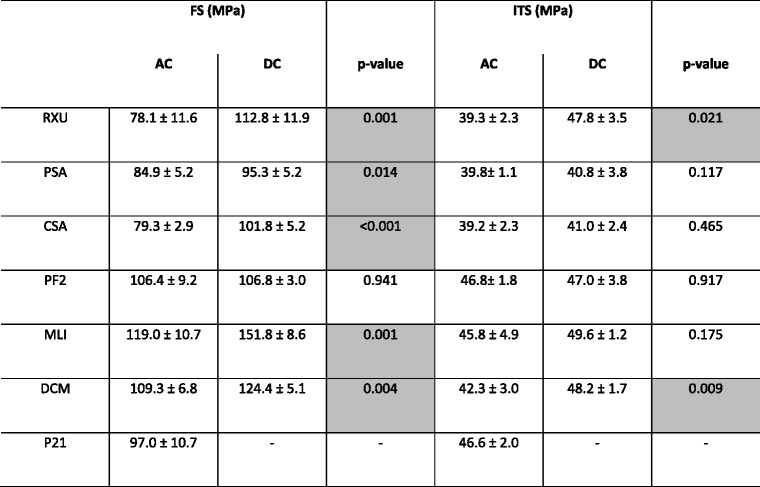
Flexural strength (FS) and indirect tensile strength (ITS) of the cements after 24 h water storage at 37 °C (mean ± standard deviation)

Significant differences (*p* < 0.05) between auto-polymerization (AC) and dual-curing (DC) are marked grey

### Indirect tensile strength tests

Shapiro-Wilk test revealed a not normal distribution for indirect tensile strength data, therefore Kruskal-Wallis was applied to test for differences between the cements. After 24 h water storage, significant differences between values obtained by dual-curing and those after auto-polymerization were only found for RXU and DCM (Table 2). All other cements attained similar values for dual-cured and auto-polymerized specimens.

Indirect tensile strength was highest for auto-polymerized specimens of PF2, P21, MLI and DCM. PSA, RXU and CSA achieved significantly lower values than the other cements (*p* < 0.001). Highest values of dual-cured specimens were achieved for MLI, DCM, RXU and PF2. Significantly lower values attained CSA and PSA (*p* < 0.001).

### Effect of water storage

**Fig. 1:**
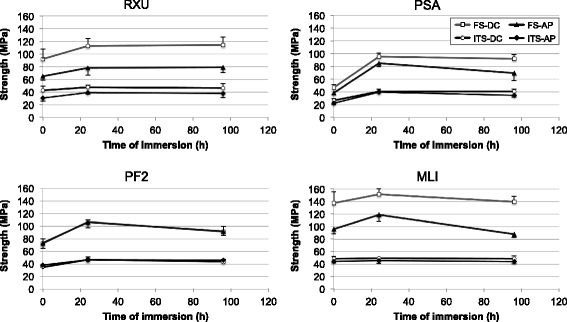
Effect of water storage at 37 °C on flexural strength (FS) and indirect tensile strength (ITS) of the respective cement after auto-polymerization (AP) or dual-curing (DC)

Flexural strength increased for all materials after 24 h of water storage compared to the state directly after curing (Fig. 1, Table 3). The increase was significant for all cements except auto-polymerized specimens of RXU and dual-cured specimens of MLI. Between 24 h and 96 h of water storage auto-polymerized specimens of MLI, PSA and PF2 as well as dual-cured specimens of PF2 exhibited a significant reduction in flexural strength.

**Table 3:**
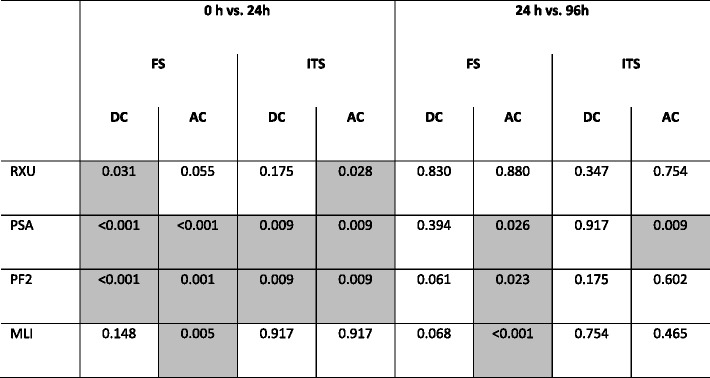
*P*-values of auto-polymerized (AP) and dual-cured (DC) cement specimens of 0 h vs. 24 h and 24 h vs. 96 h

Significant differences (*p* < 0.05) are marked grey

Indirect tensile strength increased in the first 24 h for auto-polymerized specimens of PSA, PF2 and RXU as well as for dual-cured specimens of PSA and PF2. A significant decrease between 24 and 96 h was only noted for PSA.

### Correlation between flexural strength and indirect tensile strength

**Fig. 2:**
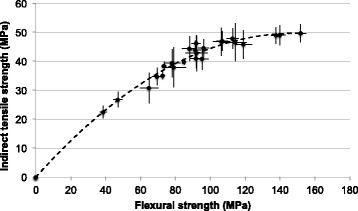
Correlation between flexural strength and indirect tensile strength of all specimens

Flexural strength and indirect tensile strength are non-linearly correlated (Fig. 2). With increasing flexural strength indirect tensile strength asymptotically approached a maximum in the range of 50 MPa.

## Discussion

The present results prove that flexural strength and indirect tensile strength are strongly correlated. However, the correlation is not linear and the indirect tensile strength test is less sensitive. Therefore, the indirect tensile strength test may only be recommended as a screening test in the early stage of material development.

### Flexural strength

Flexural strength has been established as a standard method for the investigation of mechanical strength of dental materials due to its reproducibility and reliable results [9, 10]. Nevertheless during this study the manufacturing process of the specimens for the flexural strength test was experienced as a time demanding process. Air bubbles due to the mixing procedure as well as failures during the polishing process might lead to unusable specimens. Therefore, the flexural strength test is not recommended as a screening test for resin composite cements. However, the flexural strength test is superior to the indirect tensile strength test for testing materials with high strength as well as for aging procedures due to its high sensitivity (Fig. 2).

Water storage at 37 °C resulted in an increase in strength for all cements (MLI, PF2, PSA, RXU) over the first 24 h. The effect can be explained by an increased conversion rate [11–13]. During the period between 24 h and 96 h values for dual-cured specimens of RXU and PSA as well as auto-polymerized specimens of RXU remained constant. These self-adhesive cements contain phosphate groups that are able to bind to water. Water uptake might result in an increase of volume and due to the development of internal compressive stress in an increase of strength.

A decrease in flexural strength values for dual-cured specimens of MLI, PF2 and auto-polymerized specimens of MLI, PF2, and PSA after a storage time of 96 h was recorded. MLI and PF2 obviously revealed a stronger susceptibility to water attack. For auto-polymerized PSA specimens an incomplete polymerization process with remaining monomers at the surface dissolving into the water over time might have been responsible for the decrease in strength values.

### Indirect tensile strength

Indirect tensile strength has been used to successfully evaluate aging of dental cement [8]. In the present study the indirect tensile strength test revealed low sensitivity to water storage at 37 °C. In the previously mentioned study [8] significantly lower values were achieved for PF2 directly after light curing (22.0 ± 3.2 MPa [8] vs 35.0 ± 1.3 MPa). After 24 h water storage at 37 °C a value of 47.0 ± 3.8 MPa was recorded which again is higher than the one found previously (40.0 ± 3.4 MPa) [8]. The difference is more pronounced directly after light curing and therefore might be attributed to the fact that in the present study no ED Primer II, which induces the polymerization [14], was applied on the mold.

### Curing mode

For all materials investigated and for both test methods the dual-curing procedure revealed better or similar strength values than auto-polymerization, confirming results found in previous studies [1, 4, 15, 16]. The effect of dual-curing resulting in higher values than auto-polymerizing was more pronounced for flexural strength than for indirect tensile strength (Table 2).

Resin based luting agents are advised to being exposed to light-curing in order to achieve a higher degree of conversion of the material [17]. A higher degree of conversion results in higher strength values, which was found for all cements after 24 h water storage except PF2. PF2 revealed similar values for both curing modes suggesting that the auto-polymerization process itself is very effective. The type of photo-initiator and functional monomer used in a cement influences the degree of conversion [18]. PF2 and PSA contain Campherquinone. While Campherquinone is most widely used in dental resin composites, it photo-initiates polymerization at a relatively low rate. Tertiary amines are therefore commonly added as co-initiators to accelerate polymerization [19, 20]. For Panavia F 2.0 a higher degree of conversion was found when the specimens were dual-cured (60 %) compared to autopolymerization (50 %) [14]. In the present study the degree of conversion of PF2 did not affect flexural strength or indirect tensile strength. MLI contains dibenzoyl peroxide as a chemical initiator. For a model dual-cured resin cement containing dibenzoyl peroxide the degree of conversion was significantly increased in the dual-curing mode [21]. Safety data sheet of RXU revealed a peroxide initiator system containing t-butylperoxy-3,5,5-trimethylhexanoat. A significantly lower degree of polymerization was found for autopolymerized specimens of RXU (11.05 ± 4.16 %) when compared to dual-cured specimens (37.27 ± 5.01 %) [22]. The low degree of polymerization could be the reason for the significant difference for flexural strength values for auto-polymerized specimens when compared to dual-cured specimens for MLI, RXU and PSA.

An increase in flexural strength values was noted for all cements subjected to water storage after 24 h, which might be due to the continuing polymerization process after the initial light curing [23, 24]. All materials achieved the initial strength requirements of a material of 50 MPa for flexural strength as described in ISO 4049.

### Correlation of flexural strength and indirect tensile strength

A non-linear correlation between indirect tensile strength and flexural strength was found for all specimens. The effect of aging was generally more pronounced on flexural strength than on indirect tensile strength (Fig. 1). This may be explained by the surface to volume ratio of the specimens. Aging over a short time affects mainly the surface area. Flexural strength specimens had a higher surface to volume coefficient than indirect tensile strength specimens and therefore react more sensitive to surface effects like water attack.

For composite resin cements with higher strength the indirect tensile strength test is not able to differentiate in the same sensitivity as the flexural strength test does.

## Conclusions

The results suggest that indirect tensile strength is recommended as screening test to evaluate a large number of specimens or materials at the same time within a limited range of compositions. For measurements requiring a higher degree of sensitivity, for assessing the effect of aging or for comparison of different brands the flexural strength test is the test method of choice.

## Abbreviations

AC: Auto-cured
CSA: Clearfil SA
DC: Dual-cured
DCM: DuoCem
FS: Flexural strength
ITS: Indirect tensile strength
MLI: Multilink Implant
P21: Panavia 21
PF2: Panavia F2.0
PSA: Panavia SA
RXU: RelyX Unicem 2 Automix

## References

[1] Attar N, Tam LE, McComb D (2003). Mechanical and physical properties of contemporary dental luting agents. J Prosthet Dent..

[2] Zhang Y, Lawn B (2004). Long-term strength of ceramics for biomedical applications. J Biomed Mater Res B Appl Biomater..

[3] Rohr N, Coldea A, Zitzmann NU, Fischer J (2015). Loading capacity of zirconia implant supported hybrid ceramic crowns. Dent Mater..

[4] Braga RR, Cesar PF, Gonzaga CC (2002). Mechanical properties of resin cements with different activation modes. J Oral Rehabil..

[5] Sunico-Segarra M, Segarra A (2015). A Practical Clinical Guide to Resin Cements.

[6] Bitter K, Paris S, Pfuertner C, Neumann K, Kielbassa AM (2009). Morphological and bond strength evaluation of different resin cements to root dentin. Eur J Oral Sci..

[7] Peutzfeldt A, Sahafi A, Flury S (2011). Dentin bonding of cements. Swiss Dent J.

[8] Blumer L, Schmidli F, Weiger R, Fischer J (2015). A systematic approach to standardize artificial aging of resin composite cements. Dent Mater..

[9] Yap AU, Teoh SH (2003). Comparison of flexural properties of composite restoratives using the ISO and mini-flexural tests. J Oral Rehabil.

[10] Chung SM, Yap AU, Chandra SP, Lim CT (2004). Flexural strength of dental composite restoratives: comparison of biaxial and three-point bending test. J Biomed Mater Res B Appl Biomater..

[11] Marchesi G, Navarra CO, Cadenaro M, Carrilho MR, Codan B, Sergo V, Di Lenarda R, Breschi L (2010). The effect of ageing on the elastic modulus and degree of conversion of two multistep adhesive systems. Eur J Oral Sci..

[12] Yan YL, Kim YK, Kim KH, Kwon TY (2010). Changes in Degree of Conversion and Microhardness of Dental Resin Cements. Oper Dent.

[13] Tarum H, Imazato S, Ehara A, Kato S, Ebi N, Ebisu S (1999). Post-irradiation polymerization of composites containing bis-GMA and TEGDMA. Dent Mater..

[14] Faria-e-Silva AL, Moraes RR, Ogliari FA, Piva E, Martins LR (2009). Panavia F: the role of the primer. J Oral Sci..

[15] Lu H, Mehmood A, Chow A, Powers JM (2005). Influence of polymerization mode on flexural properties of esthetic resin luting agents. J Prosthet Dent..

[16] Ilie N, Simon A (2012). Effect of curing mode on the micro-mechanical properties of dual-cured self-adhesive resin cements. Clin Oral Invest..

[17] Saskalauskaite E, Tam LE, McComb D (2008). Flexural strength, elastic modulus, and pH profile of self-etch resin luting cements. J Prosthodont..

[18] Oguri M, Yoshida Y, Yoshihara K, Miyauchi T, Nakamura Y, Shimoda S, Hanabusa M, Momoi Y, Van Meerbeek B (2012). Effects of functional monomers and photo-initiators on the degree of conversion of a dental adhesive. Acta Biomater..

[19] Stansbury JW (2000). Curing dental resins and composites by photopolymerization. J Esthet Dent.

[20] Tay FR, King NM, Suh BI, Pashley DH (2001). Effect of delayed activation of light-cured resin composites on bonding of all-in-one adhesives. J Adhes Dent.

[21] Moraes RR, Faria-e-Silva AL, Ogliari FA, Correr-Sobrinho L, Demarco FF, Piva E (2009). Impact of immediate and delayed light activation on self-polymerization of dual-cured dental resin luting agents. Acta Biomater..

[22] Vrochari AD, Eliades G, Hellwig E, Wrbas KT (2009). Curing efficiency of four self-etching, self-adhesive resin cements. Dent Mater..

[23] Janda R, Roulet JF, Latta M, Rüttermann S (2006). The effects of thermocycling on the flexural strength and flexural modulus of modern resin-based filling materials. Dent Mater..

[24] Duymus ZY, Yanikoğlu ND, Alkurt M (2013). Evaluation of the flexural strength of dual-cure composite resin cements. J Biomed Mater Res B Appl Biomater.

